# Coupling Light into Memristors: Advances in Halide Perovskite Resistive Switching and Neuromorphic Computing

**DOI:** 10.1002/smtd.202500089

**Published:** 2025-04-25

**Authors:** Zijian Feng, Jintao Wang, Fandi Chen, Beining Dong, Xinyu Ma, Tingting Mei, Ni Yang, Xinwei Guan, Long Hu, Chun‐Ho Lin, Zhi Li, Tom Wu, Dewei Chu

**Affiliations:** ^1^ School of Materials Science and Engineering University of New South Wales (UNSW) Sydney NSW 2052 Australia; ^2^ School of Science RMIT University Melbourne VIC 3000 Australia; ^3^ Department of Applied Physics The Hong Kong Polytechnic University Kowloon Hong Kong 999077 China

**Keywords:** light‐coupling, memory, optoelectronics, perovskite, resistive switching

## Abstract

Resistive switching memristor is an emerging nonvolatile memory technology designed to overcome the physical limitations of conventional systems and the performance bottleneck of the von Neumann architecture. Notably, halide perovskite (HP)‐based memristors have gained significant attention in recent years due to their unique ionic migration behavior and exceptional photoelectric properties. This review highlights HP‐based resistive switching, focusing on its recent developments in coupling light into memristors and discussing its implications for neuromorphic computing. The mechanisms of resistive switching are explored alongside the role of HP photoelectric properties in enhancing switching dynamics. The advantages and applications of light‐coupled resistive switching, including reduced switching voltage, enhanced operation reliability, multilevel switching capability, and the development of light‐integrated artificial synapses are discussed comprehensively. By fully harnessing the exceptional optoelectronic properties of HPs, this emerging field may pave the way for innovative approaches to memory technologies and light‐responsive neuromorphic systems.

## Graphical abstract

This paper comprehensively reviews the recent developments in coupling light into halide perovskite‐based memristor devices and discusses its advancements in neuromorphic computing. The mechanisms, advantages, and applications of light‐coupled resistive switching are thoroughly discussed with perspective outlooks, providing deep insight into memory technologies and light‐responsive neuromorphic systems.

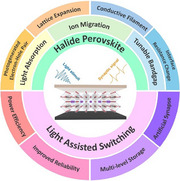

## 1 Introduction

Since the invention of the computer, memory has been a vital component in data storage and processing. Unlike conventional volatile memory that necessitates continual refreshing to prevent data loss, nonvolatile memory has garnered increasing attention for its ability to retain data permanently even without power connection. Typical nonvolatile memories such as fast large area scan hardware (FLASH) memories have been successfully scaled down to the nm level, resulting in substantial storage density and enhanced performance. However, the quantum tunneling induced leaking current becomes inevitable with the size reaching the Fermi wavelength of electrons, therefore bringing in significant challenges to further scaling down the silicon‐based storage devices.^[^ ^1^ ^]^ Besides the limitation in scaling, the energy consumption of devices is another pressing issue, driven by the increasing need for big data storage due to the recent progress in artificial intelligence (AI). For storage devices, the energy consumption arises from the high voltage required for erase and write operations, which involve the charging and discharging of capacitors within memory cells. Currently, FLASH memory typically operates at 5 V, while voltages below 0.5 V are deemed unreliable due to the limitations imposed by the Fowler–Nordheim tunneling conduction mechanism.^[^ ^2^ ^]^ As a result, a new form of memory adopting different physical mechanisms that can operate at lower voltage is needed for further improvement in energy efficiency. In order to overcome these challenges, various types of random‐access memory (RAM) have emerged, including ferroelectric RAMs, magnetic RAMs, phase‐change RAMs, and resistive switching RAMs (RRAMs).^[^ ^2^ ^]^ Among them, RRAMs possess the advantages of low power consumption, fast response speed, and the ability to achieve minimal cell sizes through crossbar matrix structures.^[^ ^2^, ^3^, ^4^, ^5^, ^6^, ^7^ ^]^ Unlike FLASH memory, which requires high voltages for Fowler–Nordheim tunneling, RRAM switches between high‐ and low‐resistance states through ion or defect migration at much lower voltages, often below 1 V, thus promising its potential for future energy‐efficient applications.

The first type of functional materials developed for RRAMs were metal oxides, after the discovery of the resistive switching (RS) phenomenon in 1962, then it was expanded to a large variety of materials. Halide perovskites (HPs) represent a more recent category of semiconducting materials utilized in RS devices. HPs have a cubic crystal structure (ABX_3_), where the flexibility of perovskite structure allows for the migration of metal ions and halide vacancies under an electric field, enabling their RS properties.^[^ ^8^, ^9^ ^]^ Their distinctive crystal structure not only facilitates the migration of ions/vacancies for RS applications but also contributes to their outstanding optoelectronic properties, which have attracted significant attention in recent years.^[^ ^10^ ^]^ HPs exhibit excellent light absorption, tunable bandgaps, high carrier mobility, and long carrier diffusion lengths, making them ideal candidates for applications in solar cells,^[^ ^11^ ^]^ light‐emitting diodes (LEDs),^[^ ^12^ ^]^ photodetectors,^[^ ^13^ ^]^ and other optoelectronic devices.^[^ ^14^ ^]^ The combination of excellent optical properties with their ability to undergo ion migration further enhances their potential in memory and RS devices. Light‐involved digital computing, enabled by optoelectronic memristors based on halide perovskites such as CH_3_NH_3_PbI_3_ and CH_3_NH_3_PbI_3−_ *_x_* Cl*_x_*, offers a promising path toward integrating sensing, memory, and logic operations in a single low‐power device. These perovskite memristors exhibit light‐responsive RS behaviors, allowing data storage and Boolean logic operations to be controlled by both electrical and optical signals.^[^ ^15^, ^16^ ^]^ The response of HPs under light stimulation can modulate the RS properties, offering a unique advantage in the development of light‐responsive or optoelectronic RS devices. These versatile applications and the optoelectronic capabilities of HPs place them at the forefront of next‐generation materials for both energy harvesting and memristor technologies.

Besides the memory function, HP RS devices can also be used for neuromorphic computing systems. The current computing landscape dominated by von Neumann architectures is hindered by high energy consumption and limited speed due to separated memory and processing units.^[^ ^17^, ^18^, ^19^, ^20^ ^]^ Recent development of HP RS devices has demonstrated their potential to act as artificial synapses by modifying the synapse weight under external electric stimuli. It is similar to the synapse behavior in biological synapses within the human brain, hence becoming the basic building blocks for neuromorphic computing systems.^[^ ^21^, ^22^, ^23^ ^]^ Different from conventional memristor synapses, HPs‐based RS devices can combine the resistance‐changing characteristics and light‐responsive properties, providing artificial synapses with higher accuracy, lower power consumption, enhanced learning and recognition capabilities, and the ability to process the light information directly.^[^ ^24^, ^25^, ^26^, ^27^ ^]^

**Figure 1:**
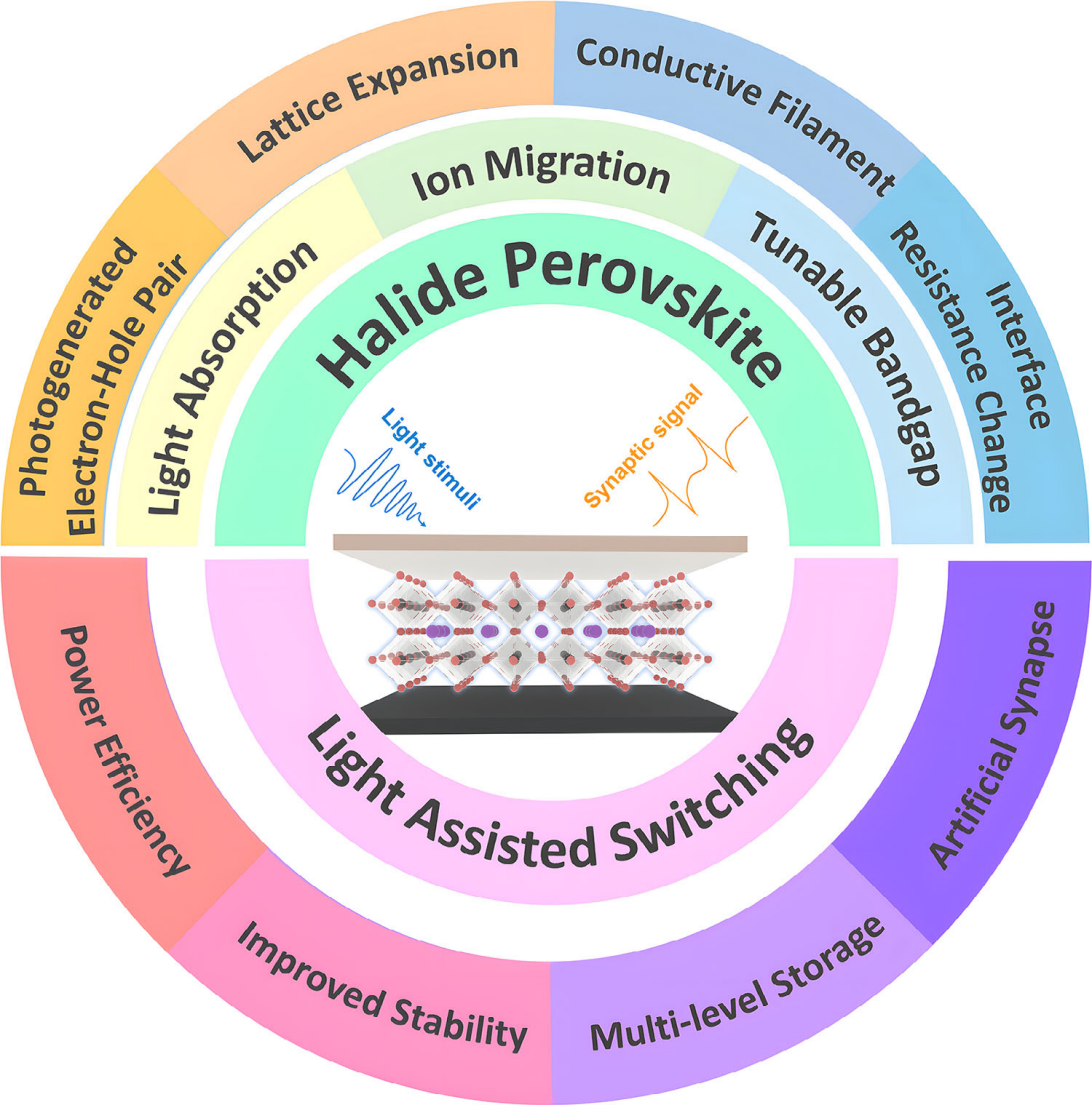
Conceptual schematic of light‐integrated HP‐based RS device, including its mechanism, characteristics, and applications.

In this review, we comprehensively discuss the concept, mechanism, advantages, and recent development of light‐coupled perovskite RS devices in memory and neuromorphic computing applications (**Figure** **1**). While most existing reviews primarily focus on electrical‐based perovskite RS devices, this review centers on integrating light into perovskite memristors, which provides an alternative perspective aiming to fully explore and harness the potential of photoresponsive HPs. The concept and mechanism of perovskite RS phenomenon are first introduced, including the device structure, key parameters, formation of conductive filament, and the change of interface resistance. Next, from the material point of view, the unique optical and electronic properties of HP are reviewed followed by the discussion on how light influences their RS characteristics, which offers a distinct opportunity to couple light and electrical signals into optoelectronic devices for advanced applications. After the discussion of the materials, the advantages and recent applications of light‐integrated perovskite RS devices in memory and neuromorphic computing are comprehensively reviewed, including the improvements in energy efficiency and operation reliability, the capability of multilevel data storage, and light‐enhanced artificial synapse application. Finally, conclusions and outlooks on light‐coupled perovskite RS devices are given, including the key challenges and potential research directions. With continuous advancement in materials and device engineering, perovskite‐based RS devices show great promise in coupling light into memristors, which may open up versatile functions in future resistive switching and neuromorphic computing applications.

## 2 Concept and Mechanism of HP‐Based RS Devices

The general layout of HP RS devices consists of a layer‐by‐layer configuration, with two electrodes sandwiching an active HP layer, whose resistance can be modulated by external electric fields. During resistive switching, the high resistance state (HRS) of the device is usually referred to as the “OFF” state, while the low resistance state (LRS) is referred to as the “ON” state.^[^ ^28^ ^]^ RRAMs utilize these two resistance states to represent “0” and “1” in the binary system. The transition from the high resistance to the low resistance is called the “Set” process, which corresponds to the “Write” process in data storage. Similarly, the switch from the low resistance to the high resistance represents the “Reset” process, commonly known as the “Erase” process in memory devices.^[^ ^4^, ^7^, ^28^ ^]^ The transition between the “ON” and “OFF” states can be classified into unipolar and bipolar switching, depending on the RS properties of the HP materials in response to the polarity of the applied voltage.^[^ ^4^ ^]^ In unipolar switching, the Set and Reset processes occur at voltages of the same polarity, while bipolar switching requires voltages of opposite polarities for the Set and Reset, often associated with ion migration or redox reactions within the active layer, with nonvolatile properties.^[^ ^2^, ^29^ ^]^ Additionally, threshold switching, sometimes referred to as monostable resistive switching, involves a transition to a low‐resistance state upon reaching a specific voltage threshold without a permanent state change, commonly used in volatile memory.^[^ ^30^ ^]^

**Figure 2:**
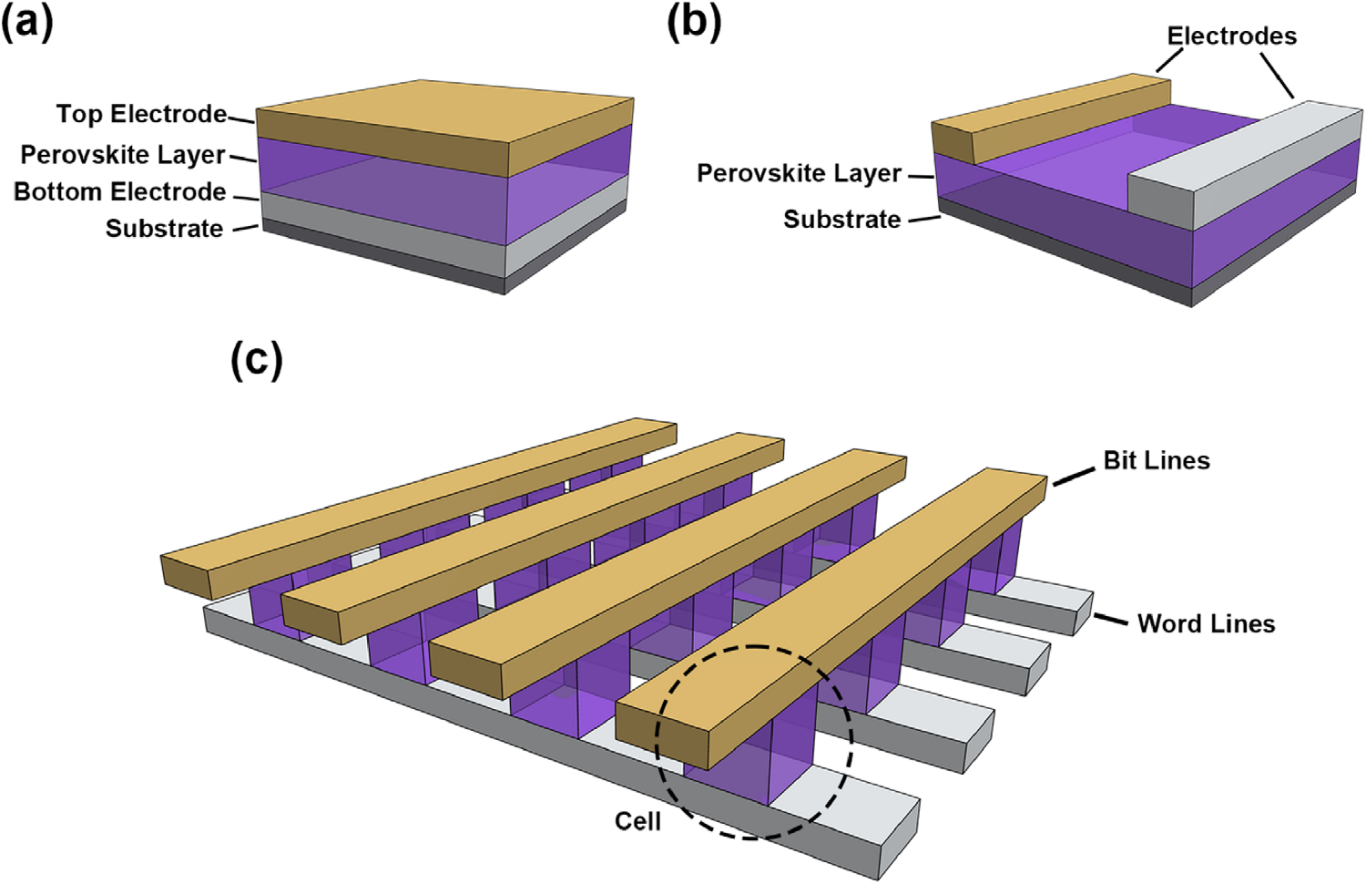
Schematic diagrams showing the layout of a) vertical MIM structure, b) planar MIM structure, and c) 3D crossbar structure.

The HP‐based RS memories are usually fabricated as individual devices adopting vertical metal–insulator–metal (MIM) structure. The vertical structure (**Figure** **2**a) is highly compatible with various thin film deposition methods, such as thermal evaporation, sputtering, and various printing and coating techniques, thus enabling high‐quality and ultrathin growth of each layer.^[^ ^31^, ^32^ ^]^ Besides the typical vertical configuration, the MIM structure can also adopt a planar layout. In planar configuration (Figure 2b), the active HP layer is placed on an insulating substrate, while two electrodes are placed on two ends of the active layer to modify the conductivity of the HP between the electrodes. The planar configuration provides a good platform to study the RS mechanism of HPs as the changes in ion/vacancy composition and corresponding distribution can be investigated during device operation. However, this structure may face challenges when fabricating active layers with short channel lengths and requires more complicated designs for device integration.^[^ ^28^ ^]^ When RS devices come into engineering application, a 3D crossbar structure or crossbar matrix can be used to integrate vertical MIM cells, as shown in Figure 2c. In such 3D configurations, the RS cells are usually integrated with another selector, such as 1T1R (transistor–resistor) or 1D1R (diode–resistor), to avoid sneak currents in the circuits. The 3D integration of RRAMs can reduce the minimum cell size to (4/*n*)*F* ^2^ (*n* is the number of stacking layers and *F* is the minimum feature size), which is one significant advantage when compared with other memory technologies.

**Figure 3:**
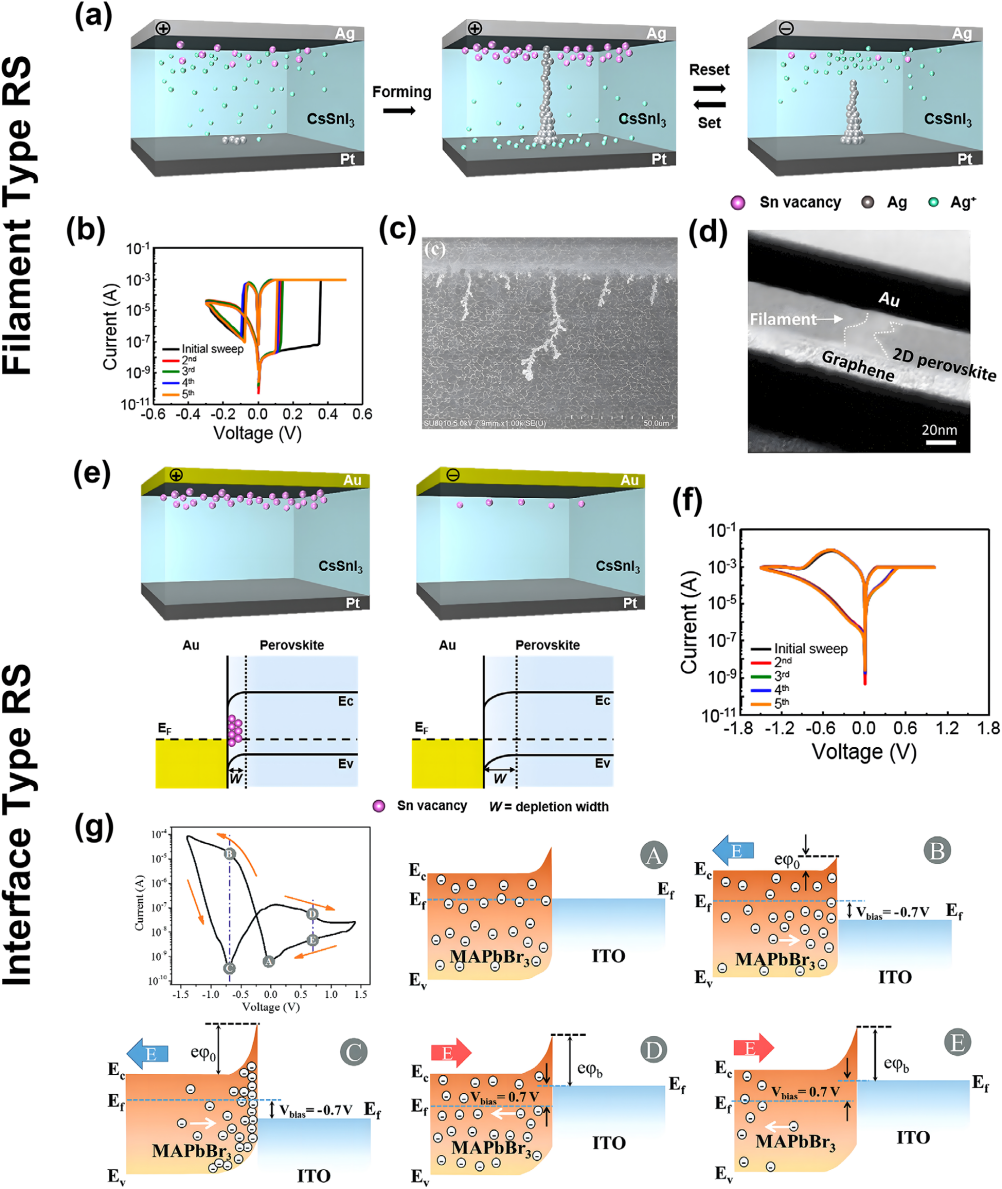
Two major switching mechanisms for HP‐based RS devices. The Conductive filament formed by Ag ion migration from the electrode into CsSnI_3_ perovskite layer, as shown in a) schematic diagram and b) corresponding hysteresis loop. (a,b) Reproduced with permission.^[^ ^34^ ^]^ Copyright 2019, American Chemical Society. c) SEM image of conductive filament formed by Ag diffusing into CsPbI_3_ perovskite layer. Reproduced with permission.^[^ ^35^ ^]^ Copyright 2020, American Chemical Society. d) The conductive filament formed by Br ions in graphene|(PEA) _2_PbBr_4_|Au device as shown in TEM image. Reproduced with permission.^[^ ^39^ ^]^ Copyright 2017, American Chemical Society. e) Schematic diagram and f) hysteresis loop of Pt|CsSnI_3_|Au device induced by the accumulation of Sn vacancies. (e,f) Reproduced with permission.^[^ ^34^ ^]^ Copyright 2019, American Chemical Society. g) Schematic diagram showing the accumulation of Br ions near the interface between perovskite and electrode. The circled A to E correspond to different procedures in the hysteresis loop. Reproduced with permission.^[^ ^43^ ^]^ Copyright 2018, Wiley‐VCH.

The tuning of resistance in HP RS devices comes from two mechanisms: the formation of conductive filament and the change of resistance at the interface. The conductive filament involves using a Set voltage to create a “shortcut” for charge flow within the high‐resistance bulk HP under specific conditions, resulting in a reduction in the device's resistance. Conversely, when the “shortcut” is disrupted under a Reset voltage, the HP material exhibits a high‐resistance state.^[^ ^33^ ^]^ Various factors are responsible for forming conductive filaments in RS devices, including thermal, electronic, and ionic effects. For HP materials, mobile ions/vacancies serve as the primary contributors to the formation of conductive filaments, which can be further classified into two mechanisms: electrochemical metallization (ECM) and the valence change mechanism (VCM). An illustration of the ECM mechanism is shown in **Figure** **3**a, where the HP (CsSnI_3_) device is combined with electrochemical active electrodes, such as Ag, in which the Ag electrode is oxidized into Ag^+^ ions migrating through the perovskite layer under external voltages and forming a conductive filament.^[^ ^34^ ^]^ In this process, the formation of conductive filament usually demonstrates a “forming” step prior to the “set” process in the hysteresis loop, as shown in Figure 3b. The scanning electron microscopy (SEM) image in Figure 3c reported by Xu et al.^[^ ^35^ ^]^ shows the formation of Ag filaments (white lines) in the CsPbI_3_ HPs layer, confirming the ECM mechanism. The ECM‐based switching is associated with the active electrodes, where the active metal can contribute ions to form conductive filaments, common materials include Ag and Cu. A different type of RS happens in perovskites when RS devices are composed of chemically inert electrodes, such as Au, Pt, and ITO. For such devices, the RS performance relies on the mobile ions within the perovskite, in which migration of halide ions or vacancies through the perovskite lattice is commonly observed.^[^ ^36^, ^37^ ^]^ Perovskites possess ionic crystal structures and mobile ions might be generated under an external field. With high electric fields, the charged halide ions/vacancies can migrate toward the electrode and form conductive filaments, known as the VCM phenomenon.^[^ ^38^ ^]^ Figure 3d demonstrates the transmission electron microscopy (TEM) images of graphene|(PEA)_2_PbBr_4_|Au RS device as an example obtained by Tian et al.,^[^ ^39^ ^]^ in which the conductive filaments were formed by Br vacancies under external voltage, and the filament can break when reset voltage is applied. A study by Yan et al. compared different metal electrodes, suggesting that with inert electrodes, the switching might be slower but more stable, relying on the perovskite's intrinsic properties.^[^ ^40^ ^]^

The interface between the HP layer and the electrode, where a Schottky barrier can be formed, is another origin of resistance change.^[^ ^4^, ^41^ ^]^ In interface‐typed RS devices, the width and height of the Schottky barrier can be modified by the accumulation of mobile ions, such as halogen ions or vacancies in HPs. These mobile ions gathering near the interface between the electrode and the perovskite layer under applied external voltages will change the width of the depletion layer, thereby changing the interface resistance,^[^ ^42^, ^43^ ^]^ as shown in Figure 3e,f. In a representative study, Han et al.^[^ ^34^ ^]^ fabricated CsSnI_3_ perovskite devices combined with chemically active Ag electrode (Figure 3a) and chemical inert Au electrode (Figure 3e), respectively. When the top electrode (TE) is switched to Au, the perovskite device can behave in a different conduction mechanism, which can be distinguished from the different shapes and switching voltage in the hysteresis curve, as shown in Figure 3f. Different from filamentary type Pt|CsSnI_3_|Ag perovskite device, Han et al.^[^ ^34^ ^]^ suggests that the resistance change in Pt|CsSnI_3_|Au is caused by Sn vacancies accumulated near the interface between the perovskite and Au, which reduces the width of the depletion layer and hence decreases the resistance. Unlike the conductive filament mechanism, the interface type resistance change happens homogeneously across the surface and exhibits a stronger dependence on the electrode contact area compared to filamentary type RS.^[^ ^44^ ^]^ Guan et al.^[^ ^43^ ^]^ investigated ITO|MAPbBr_3_|Au devices (Figure 3g), highlighting the hysteresis loop alongside a schematic diagram illustrating the relationship between perovskite ions and the barrier height at the interface between the perovskite layer and ITO electrode. When a negative voltage is applied to the device, the Br ions migrate toward the boundary of ITO electrode, increasing the barrier height. Conversely, under a positive applied voltage, the Br ions migrate away from the ITO electrode and reduce the barrier height. Kim et al.^[^ ^45^ ^]^ further explored the accumulation of ions and vacancies near the interface using energy dispersive X‐ray spectroscopy (EDS) line mapping. In the region ≈0.1–0.2 µm from the Al–MAPbI_3_ interface, a significant increase in iodine content was observed following the application of a 5 V external voltage, providing direct evidence of halogen migration and accumulation under applied electric fields. For devices using more than one perovskite or semiconductor material forming heterojunctions, the change in resistance could also happen at the heterojunction interface with proper designs.^[^ ^46^ ^]^

## 3 Optoelectronic Properties of HP Materials

Up to date, various functional materials have been investigated and used for RS applications. Among them, HP shows exceptional optoelectronic properties, including tunable bandgaps, high optical absorption coefficients, and efficient charge transport characteristics, thus benefiting optical‐dependent RS applications.^[^ ^47^ ^]^ With a high absorption coefficient exceeding 10^5^ cm^−1^, HPs exhibit incredible light‐absorbing properties, enabling efficient photon harvesting even in ultrathin films.^[^ ^48^ ^]^ This impressive absorption is attributed to their direct bandgap, unique electronic band structures, high defect tolerance, and reduced exciton binding energy.^[^ ^49^, ^50^ ^]^ Beyond their absorption properties, the tunability of the optical bandgap is a crucial feature. In optoelectronic devices, optimizing device performance and tailoring it for various applications often require tuning the material bandgap to match the desired wavelength. In this regard, HPs, offering highly tunable bandgaps and outstanding optoelectronic properties, provide an ideal platform for versatile applications. The high tunability of perovskites’ optical bandgap arises from the varied electron coupling influenced by adjustments to their crystal structure and composition. HPs form their band structures with major effects from B‐site and X‐site electron orbit.^[^ ^51^, ^52^ ^]^ Huang and Lambrecht demonstrated that the band structure near the Fermi surface is predominantly determined by the outermost p‐orbitals of the B‐site cations and halide ions.^[^ ^52^ ^]^ Moreover, the change in band structure as a result of orbital coupling can lead to adjustable absorbance and photoluminescence properties. Since the perovskite structure can accommodate a wide variety of B‐site cations and X‐site anions, its properties can be effectively tuned to meet the specific requirements of diverse optoelectronic applications.

**Figure 4:**
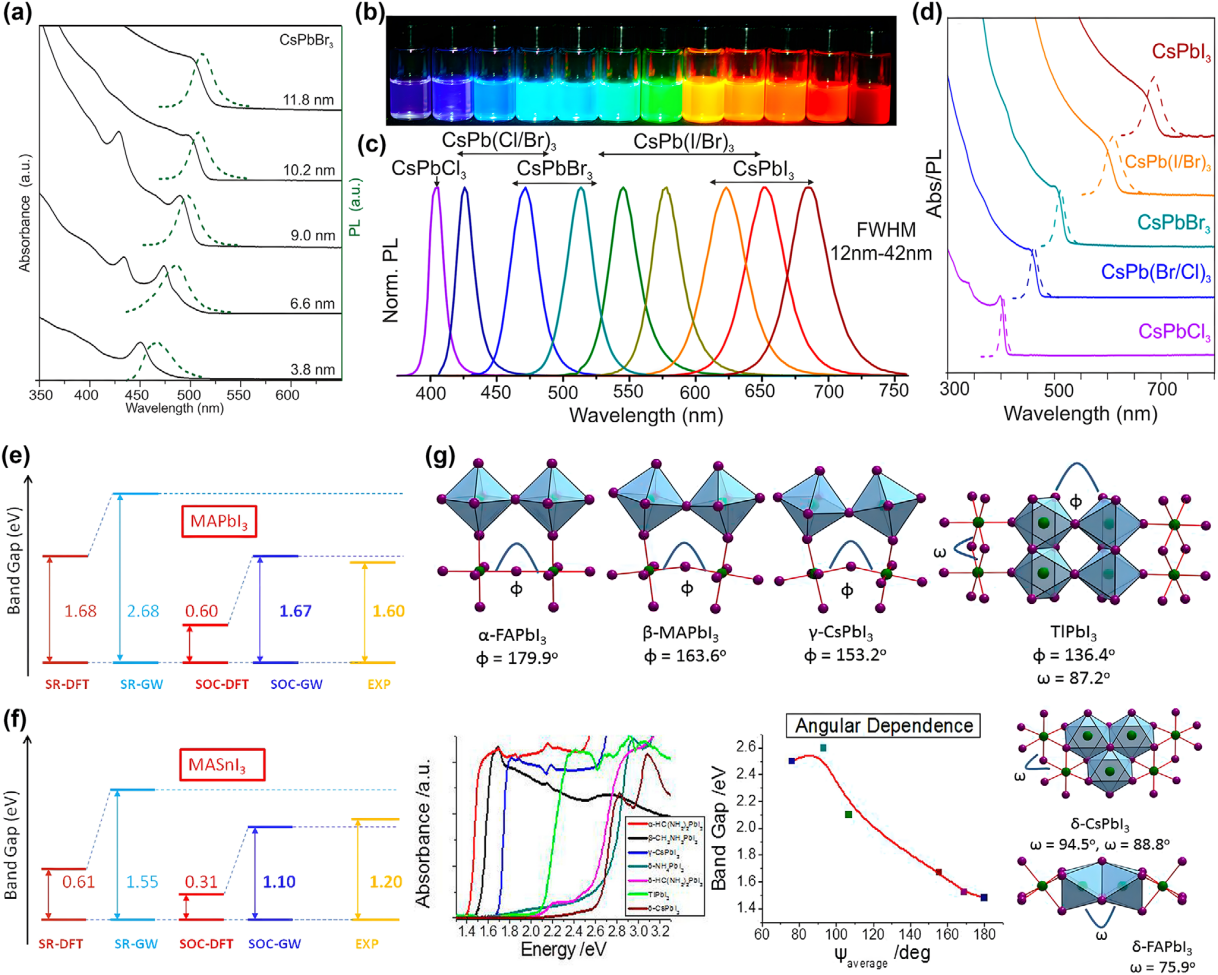
a) Optical property changes in absorption and PL spectra with different‐sized CsPbBr_3_ nanocrystals. CsPbX_3_ (X = Cl, Br, and I) halide perovskite nanocrystals exhibit b) different light luminescence under UV lamp and varied c) PL spectra and d) optical absorption spectra. (a–d) Reproduced with permission.^[^ ^51^ ^]^ Copyright 2015, American Chemical Society. e,f) Comparison of DFT simulated and experimental obtained bandgap between e) MAPbI_3_ and f) MASnI_3_. (e,f) Reproduced with permission.^[^ ^54^ ^]^ Copyright 2014, Springer Nature. g) Bond angles affected by perovskite A‐site cations and corresponding changes in the bandgap and absorption properties. Reproduced with permission.^[^ ^55^ ^]^ Copyright 2015, American Chemical Society.

Protesescu et al.^[^ ^51^ ^]^ studied the effect of nanocrystal size and halide composition on the optical properties of CsPbX_3_ (X = Cl, Br or I) nanocrystals. As shown in **Figure** **4**a, with the average size of nanocrystal increases from 3.8 to 11.8 nm, a significant red shift in absorbance and photoluminescence peak is observed, indicating the optical properties tuned via modifying the crystal size. By employing halide composition engineering (Figure 4b), the perovskite colloidal solutions can also feature different photoluminescence (PL) emission color under UV lamp, correlated with the PL and optical absorption spectra in Figure 4c,d. Starting with the CsPbBr_3_, increasing the content of iodine will cause a red shift in PL and absorption peak of perovskites, and increasing the content of chloride will shift the PL peak toward a smaller wavelength. Similar trends have also been observed in CsPbX_3_ by Aharon and Etgar,^[^ ^53^ ^]^ who studied the effects of X‐site halide composition on double perovskite (OA)_2_(MA)_2_Pb_3_X_10_ (X = Br, I) devices. With the increasing bromide content, the absorption and PL peaks shift to lower wavelengths, corresponding to larger energies, similar to the result in CsPbX_3_. Umari et al.^[^ ^54^ ^]^ studied the impact of altering the B‐site cation by comparing the MAPbI_3_ and MASnI_3_, as shown in Figure 4e,f, revealing that the bandgap decreased when replacing Sn with Pb, consistent with both density‐function theory (DFT) simulation and experiment‐measured results. Although the electron orbitals of A‐site cations generally have less impact on the band structure, altering the A‐site cations can still modulate the bandgap and absorption properties by influencing the bond angles in the perovskite lattice through variations in ionic radii. As shown in Figure 4g, Stoumpos and Kanatzidis^[^ ^55^ ^]^ compare the bond angle of different APbI_3_ perovskites, where A‐site cations include FA group, MA group, Cs, and Tl group. It was found that both absorbance property and bandgap are highly dependent on the bond angle between B‐site and X‐site ions (B–X–B angle or Φ), which varies with changes in the A‐site cation. These studies collectively highlight the versatility of HPs in tailoring optoelectronic properties through precise control over their composition and structure, making them promising candidates to couple light signals into memristor systems for advanced optoelectronic data processing and neuromorphic computing.

**Figure 5:**
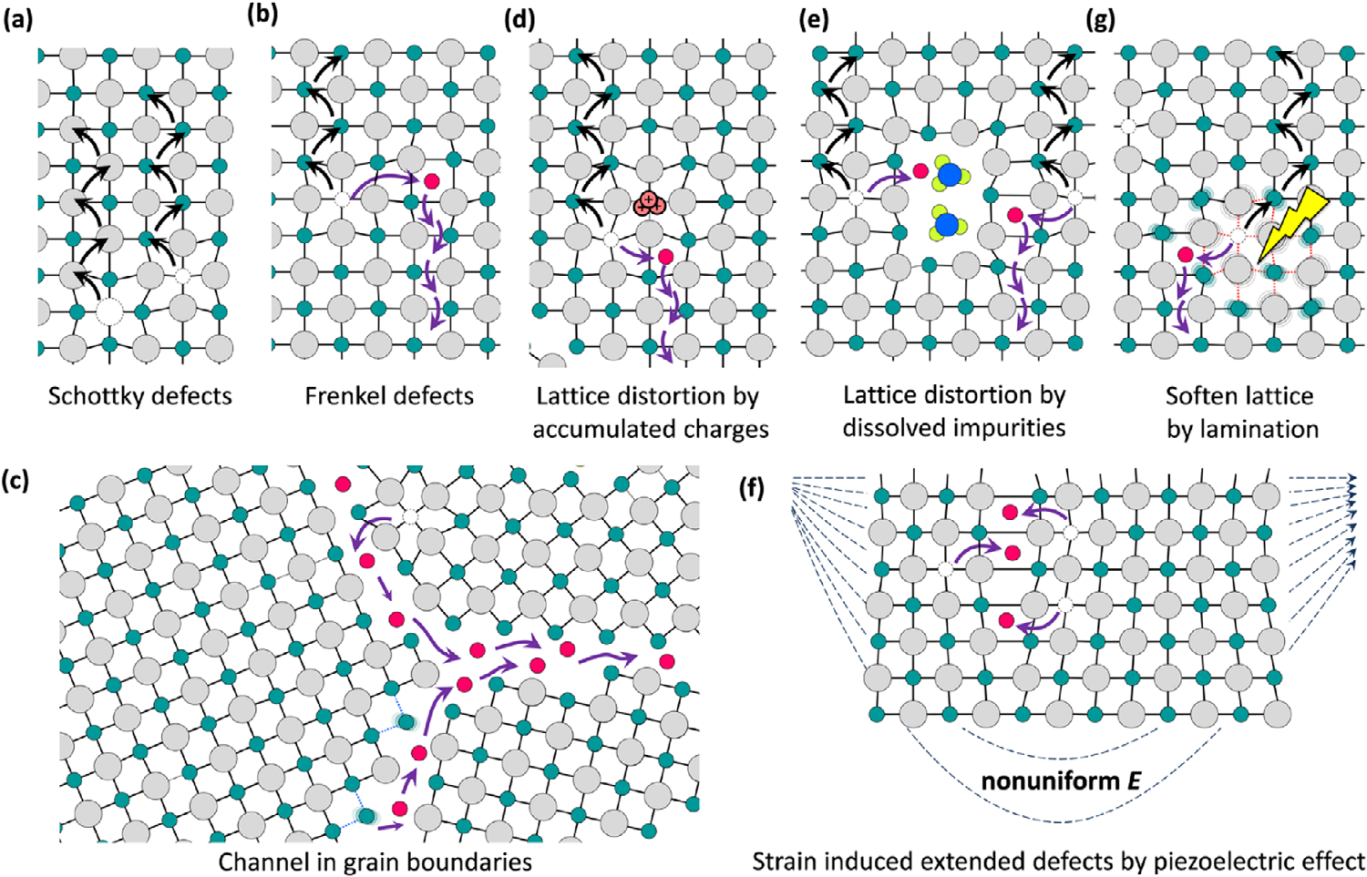
Summarized ion migration mechanism in halide perovskite lattices via a) Schottky defects, b) Frenkel defects, c) grain boundaries, d) lattice distortion caused by charge accumulation, e) lattice distortion caused by impurities, f) piezoelectric effect, and g) light illumination. (a–g) Reproduced with permission.^[^ ^62^ ^]^ Copyright 2016, American Chemical Society.

In regard of HPs’ electronic properties, an important feature is their ionic crystal lattice that allows ion migration to happen, thus featuring distinct electronic–ionic dual transports. Moreover, the unique ion migration properties of perovskites can assist in the formation of conductive filament, or forming charge accumulation layer near the interface, making them promising candidates in RS‐associated applications. Ion migration in perovskites, a critical factor influencing their electronic performance, occurs through several distinct pathways. A glimpse of the perovskite ion migration mechanism is shown in **Figure** **5**. For typical perovskites such as MAPbI_3_, internal factors such as Schottky defects represent a fundamental ion migration mechanism involving paired vacancies of cations and anions, such as MA^+^ and PbI_3_^−^, which form without creating significant electronic trap states.^[^ ^56^ ^]^ This preserves charge transport efficiency by maintaining a defect‐tolerant lattice, as shown in Figure 5a. Frenkel defects add another dimension, wherein cations like Pb^2+^ move to interstitial positions.^[^ ^57^ ^]^ These defects act as unintentional dopants, influencing electronic properties while enabling localized ion migration pathways, as shown in Figure 5b. Grain boundaries provide prominent migration pathways due to their structurally disordered regions and weak bonding, which lower migration energy barriers (Figure 5c). These pathways dominate polycrystalline films, where grain interfaces act as conduits for ionic drift, particularly under an applied electric field.^[^ ^58^ ^]^ Impurities, shown in Figure 5e, often residues from precursor solvents or unreacted species, distort the lattice and create localized regions of low structural coherence, further facilitating ion transport.^[^ ^59^ ^]^

On the external factors, charge accumulation under external electric field,^[^ ^60^ ^]^ and the nonuniform electric fields can induce nonuniform strain via the piezoelectric effect,^[^ ^61^ ^]^ which is often observed on perovskite single crystals, as shown in Figure 5d,f, creating local dislocations and defects that enable ion migration. Under illumination, light‐induced lattice softening occurs, weakening bonds through photoinduced carrier generation. This process facilitates ion migration by reducing activation barriers for ion movement,^[^ ^62^ ^]^ as shown in Figure 5g, and will be further discussed in the following section. In short, the abundant ionic pathways in HP lattices position them as promising candidates for resistive switching memristors, while their superior optical properties enable the integration of light signals into electronic data processing, thus paving the way for energy efficient and high performance optoelectrical neuromorphic computing.

## 4 The Influence of Light on Perovskite RS Properties

**Figure 6:**
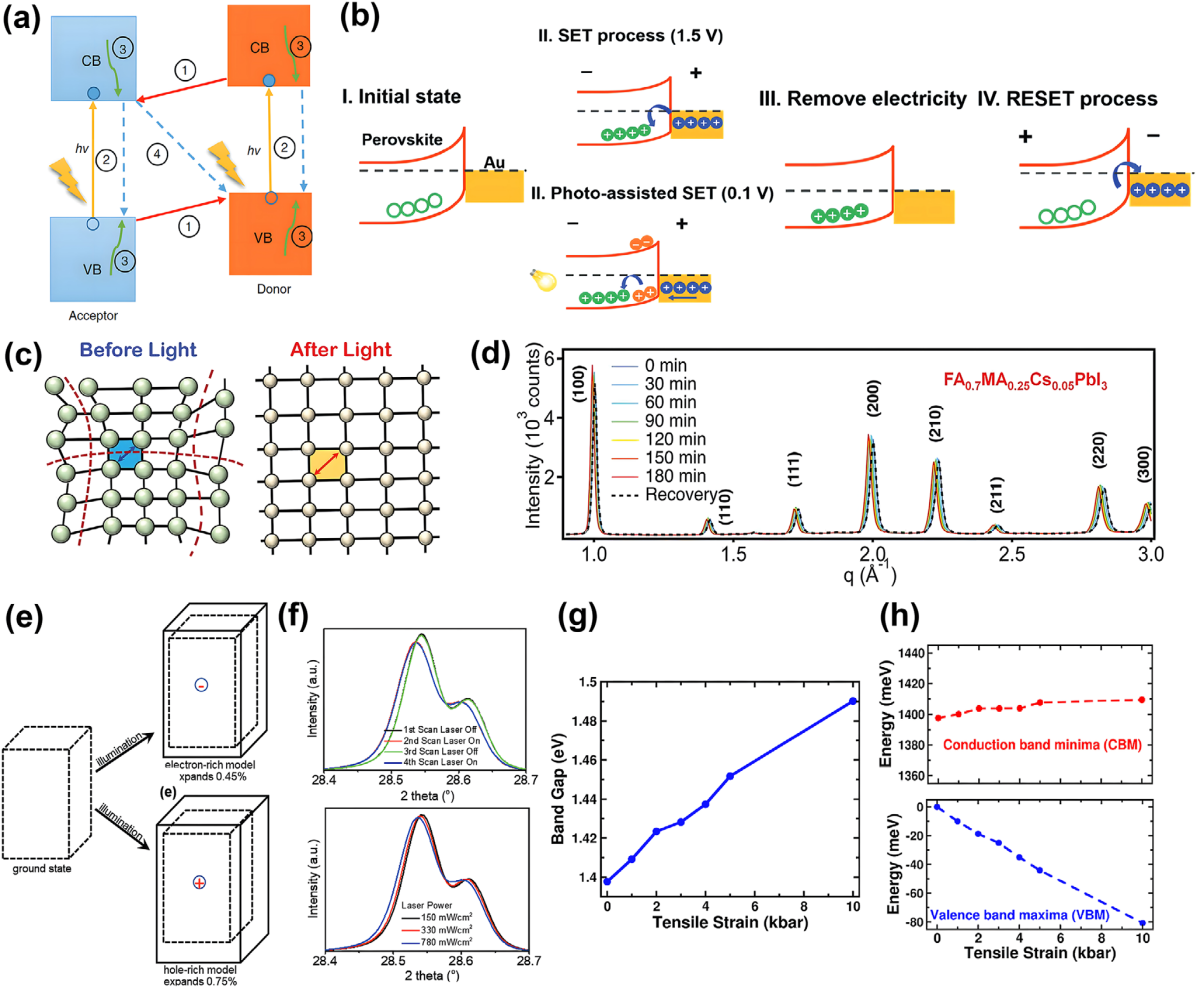
a) The photogenerated electron–hole pairs related to the electronic energy levels. The labels represent ① electron or hole transfer, ② electron and hole recombination inside materials, ③ electron and hole relaxation, and ④ charge recombination at interface. Reproduced with permission.^[^ ^65^ ^]^ Copyright 2017, John Wiley & Sons. b) Schematic diagrams about the flow of electrons and holes affecting the band structure of FTO|MAPbI_3−_ *_x_* Cl*_x_* |Au device. Reproduced with permission.^[^ ^15^ ^]^ Copyright 2018, Wiley‐VCH. c) Schematic diagram of lattice expansion under light illumination. d) GIWAXS mapping showing the change in the lattice constant of FA_0.7_MA_0.25_Cs_0.05_PbI_3_ perovskite film under different illumination times. (c,d) Reproduced with permission.^[^ ^71^ ^]^ Copyright 2018, American Association for the Advancement of Science (AAAS). e,f) Lattice expansion calculated by the DFT method in electron‐rich and hole‐rich models, respectively, and the shift in XRD peak due to MAPbI_3_ lattice expansion under laser. Reproduced with permission.^[^ ^72^ ^]^ Copyright 2019, Wiley‐VCH. g,h) Changes of perovskite bandgap with the increase of tensile strain introduced by lattice expansion. Reproduced with permission.^[^ ^74^ ^]^ Copyright 2019, American Chemical Society.

Compared to conventional materials used in RS devices, one of the outstanding properties of HPs is their exceptional response to light, enabling photosensitive RS applications. The mechanism of light‐assisted RS is described via the formation of photoexcited electron–hole pairs and the change of perovskite crystal lattice under light. When a perovskite film is exposed to light of a specific wavelength, with its corresponding energy larger than the bandgap, electrons at the top of the valence band will absorb the energy and jump to the conduction band, leaving behind positively charged holes in the valence band, thus creating photoexcited electron–hole pairs.^[^ ^63^, ^64^ ^]^ As illustrated in **Figure** **6**a, the photogenerated electron–hole pairs undergo recombination and relaxation within the material, as well as recombination at interfaces.^[^ ^65^ ^]^ In an asymmetric device structure, the photogenerated electrons tend to migrate toward the electrode with a lower work function, while the holes move toward the electrode with a higher work function, hence forming an open‐circuit voltage.^[^ ^15^, ^37^, ^66^, ^67^ ^]^ Thus, HPs exhibit varied photovoltaic properties, contributing to diverse optoelectronic applications, like photocatalysts,^[^ ^68^ ^]^ solar cells,^[^ ^69^ ^]^ and photodetectors.^[^ ^13^, ^70^ ^]^ This phenomenon can affect the resistance properties by modulating the Schottky‐like barrier at the interface between the perovskite layer and electrode.^[^ ^15^, ^37^, ^66^, ^67^ ^]^ An example of this phenomenon has been demonstrated in the FTO|MAPbI_3−_ *_x_* Cl*_x_* |Au device,^[^ ^15^ ^]^ as shown in Figure 6b. In the initial state, a Schottky barrier is formed between the Au electrode and perovskite layer due to the difference in their work functions, corresponding to the HRS of the device. During the typical electrical SET process, the holes are injected from the Au electrode into the perovskite, overcoming the Schottky barrier under external voltages. The holes trapped in the perovskite layer shifted the Fermi level of perovskite, reducing the Schottky barrier and switching the device into LRS. For the photoassisted SET process, in contrast, the photogenerated electron–hole pairs can be separated by external voltages, leading to the reduction of the Schottky barrier prior to the hole injection from the electrode. Hence the photoassisted SET voltage is significantly lower than the switching voltage without light illumination, which can greatly improve the energy‐efficiency.

Besides the effect from photogenerated electron–hole pairs, the change in lattice structure under light stimulation also contributes to the change in RS behavior.^[^ ^71^, ^72^ ^]^ Tsai et al.^[^ ^71^ ^]^ reported the lattice expansion of cubic structural FA_0.7_MA_0.25_Cs_0.05_PbI_3_ perovskite using a single light source of 1‐sun full‐spectrum (100 mW cm^−2^). As shown in Figure 6c, the schematic diagram demonstrates the expansion in the crystal lattice, and Figure 6d is the grazing‐incidence wide‐angle X‐ray scattering (GIWAX) result, which was used to study the change in lattice structure. The peaks of scattering angle *q* for the perovskite thin film shift toward the left and become more significant when illumination time increases. The shift in scattering angle represents the increase in isotropic increase in lattice constant and proves the expansion of the lattice.^[^ ^71^ ^]^ Liu et al.^[^ ^72^ ^]^ also studied the change in the lattice structure of MAPbI_3_ under light illumination with DFT calculation (Figure 6e), in which expansions of 0.45% and 0.75% in the crystal lattice were observed in electron‐rich and hole‐rich models, respectively. The X‐ray diffraction (XRD) experiment of MAPbI_3_ with and without 640 nm laser illumination (Figure 6f) revealed the peak shifts in the (220) and (004) planes under laser exposure, indicating lattice expansion. This shift is reversible when the laser is turned off and becomes more pronounced when increasing the intensity of 640 nm laser illumination. The expansion in lattice provides higher mobility for charge carriers, like halide ions or vacancies, and hence assists the conductive filament formation in RS behavior. As a result, ion migration and conductive filament formation are promoted with light coupling, leading to a reduced RS SET voltage and improved energy efficiency.

Other studies revealed that the expansion in the perovskite lattice can also affect the bandgap and charge carrier lifetime, and hence affect the electronic properties.^[^ ^73^, ^74^ ^]^ Ghosh et al.^[^ ^74^ ^]^ using computation method, conducted a study on the band structure of FAPbI_3_ perovskite under lattice expansion, observing significant changes in its crystal structure, optoelectronic properties, and charge carrier dynamics. As illustrated in Figure 6g,h, under lattice expansion, there is a noticeable reduction in the valence band maximum while the conduction band minimum remains almost unchanged, indicating an increase in the bandgap. This band structure variation can accordingly alter the Schottky barrier height at the perovskite/electrode interface, which offers opportunities to regulate the resistance state of interface‐typed perovskite RS memristors. Kim and Hagfeldt^[^ ^73^ ^]^ also suggest that under light coupling, the elongation of Pb─I bonds results in reduced octahedral tilting, leading to a more symmetric lattice structure, which may decrease the number of trap sites. The decreased trap site can reduce the charge‐induced recombination in the perovskite, hence improving the photocarrier gain and altering the resistance state of perovskite devices. In summary, the key properties of perovskites that respond to light, such as the generation of photogenerated electron–hole pairs and the enhanced ion migration, can both play significant roles in the RS mechanism, including the formation of conductive filament and change in interface resistance. By leveraging the superior optoelectronic properties of perovskite, these light‐induced effects can modify the properties of RS devices, not only enhancing their performance and functionality but also enabling the processing of light signals for advanced applications.

## 5 Advantages of Light‐Integrated Perovskite RS Devices

### 5.1 Reduction in Switching Voltage

**Figure 7:**
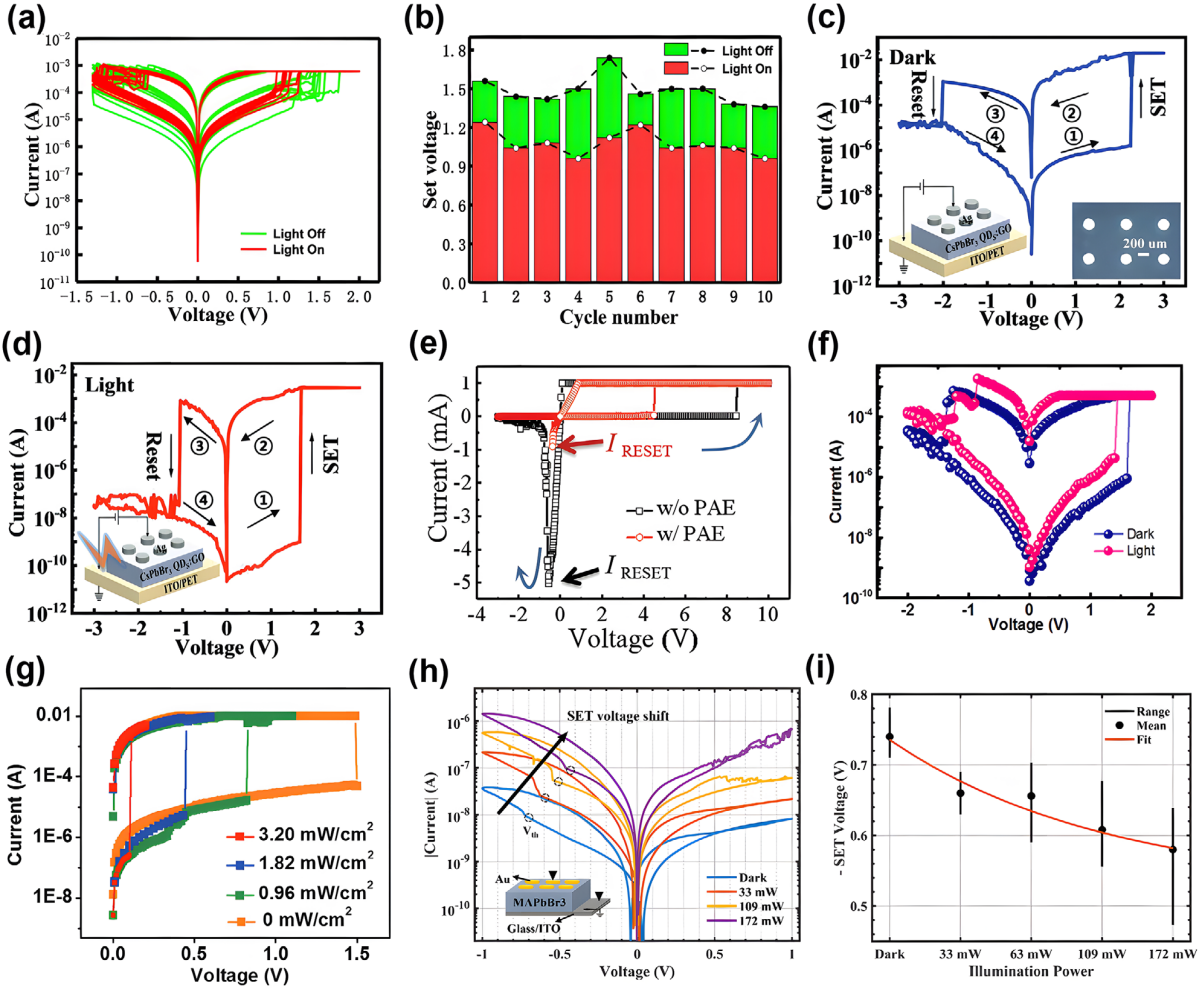
a,b) Changes in switching voltage for ITO|PMMA|CsPbBr_3_|PMMA|Au RS device with and without light illumination for 10 cycles. Reproduced with permission.^[^ ^25^ ^]^ Copyright 2019, IOP Publishing. c,d) Reduced switching voltage under light coupling for ITO|CsPbBr_3_:GO|Ag device. Reproduced with permission.^[^ ^75^ ^]^ Copyright 2022, Wiley‐VCH. e) Changes in switching voltage for FTO|MAPbI_3_|Al device when applying the PAE method. Reproduced with permission.^[^ ^76^ ^]^ Copyright 2020, Wiley‐VCH. f) Changes in switching voltage under light coupling for ITO|FAPbBr_3_|Al device. Reproduced with permission.^[^ ^77^ ^]^ Copyright 2023, Elsevier. g) FTO|MAPbI_3−_ *_x_* Cl*_x_* |Au device showing the reduction in set voltage when increasing the power density of white LED from 0 to 3.20 mW cm^−2^. Reproduced with permission.^[^ ^15^ ^]^ Copyright 2018, Wiley‐VCH. h,i) ITO|MAPbBr_3_|Au device showing a reduction in operating voltage with the increase of illumination level from dark condition to 172 mW (405 nm LED). Reproduced with permission.^[^ ^79^ ^]^ Copyright 2023, American Chemical Society.

The reduction in switching voltage is one of the most reported advantages for light‐integrated RS devices. The mechanisms involve photogenerated electron–hole pairs, which enhance conductivity and lattice expansion and further promote the migration of ionic carriers. These two effects lowered the voltage required for the transition between HRS and LRS, thereby enabling operation at reduced voltages and significantly decreasing the energy consumption of the RS device. In 2018, Zhang et al.^[^ ^25^ ^]^ investigated the resistive switching properties of CsPbBr_3_ nanocrystals (NCs) embedded in polymethylmethacrylate (PMMA) layers. The device was constructed with an ITO bottom electrode (BE) and an Au TE, forming a lamellar structure of Au|PMMA|PMMA:CsPbBr_3_‐NCs|PMMA|ITO, which exhibited bipolar switching with an On/Off ratio of 100, as shown in **Figure** **7**a. Various wavelengths were used to measure the photoresponse of the CsPbBr_3_, and a photocurrent of 2.0 nA was recorded under 365 nm light. Under light coupling (Figure 7b), a reduction in switching voltage of 0.3–0.5 V was observed during 10 Set and Reset cycling tests, demonstrating a light‐tunable resistive switching behavior. A similar phenomenon has been observed in the CsPbBr_3_ device with an Ag TE. Liu et al.^[^ ^75^ ^]^ studied the light responsivity of ITO|CsPbBr_3_ quantum dots (QDs): GO|Ag device (Figure 7c), in which the device behaves a typical bipolar digital switching, with a set voltage of 2.28 V and reset voltage −2.04 V. Under light illumination, the set voltage and reset voltage are reduced to 1.68 and −1.08 V respectively, as shown in Figure 7d. The examples mentioned above demonstrate reduced power consumption due to lower operating voltages, however, the need for continuous light irradiation may partially offset this advantage. To address this problem, in 2020, Zhao et al.^[^ ^76^ ^]^ proposed a photoassisted electroforming (PAE) method, which involves introducing light irradiation only during the initial electroforming process of the conductive filament, rather than throughout the entire switching process. Using FTO|MAPbI_3_|Al devices, they applied light with a power output of 150 mW cm^−2^ at wavelengths of 415 and 543 nm, as demonstrated through comparative tests shown in Figure 7e. The device switching voltage decreased as the light source power increased up to 150 mW cm^−2^ and remained unchanged beyond this value. Both 415 and 543 nm wavelengths exhibit similar behavior as their corresponding energies both exceed the bandgap of MAPbI_3_. With the assistance of the PAE method, the voltage required for conductive filament formation was reduced by half. As shown in the Figure 7f, Siddik et al.^[^ ^77^ ^]^ also reported a reduction in operation voltage for FAPbBr_3_ perovskite RS devices in 2022. This device featured a FAPbBr_3_ perovskite layer with an Al TE and an ITO BE, achieving bipolar switching through the formation of conductive filaments by Al atoms and Br vacancies. Under illumination with a 405 nm light source at 30 mW cm^−2^, the set voltage decreased from +1.58 to +1.32 V, and the reset voltage decreased from −1.31 to −0 .91 V. Additionally, a change in the current levels of the HRS and LRS was observed under light exposure, indicating potential applications in multilevel switching. Zhao et al.^[^ ^76^ ^]^ also suggests that the PAE method can improve the operation reliability of RS devices, which will be discussed in the following section. The intensity of light can also affect the reduction of switching voltages. As shown in Figure 7g, Zhou et al.^[^ ^15^ ^]^ studied the responsiveness of FTO|MAPbI_3−_ *_x_* Cl*_x_* |Au hybrid perovskite devices to white LED light. When the device is placed under dark condition, a 1.47 V external voltage is required to make the transition from HRS to LRS, and the On/Off ratio is ≈10^3^. When increasing the power density of the light source to 3.20 mW cm^−2^, the set voltage is reduced to 0.1 V, and the HRS current level is also reduced, corresponding to lower energy consumption and a larger On/Off ratio. Similarly, Yadav et al. studied the ITO|MAPbBr_3_|Au device response to 405 nm LED light with different power outputs, as shown in Figure 7h,i. With the light source increase from 0 to 172 mW, the average set voltage reduced from 0.74 to 0.58 V, showing the capability to reduce operation voltage with light coupling. Besides the direct influence on SET and RESET voltage, other studies also investigated the relationship between light coupling wavelength and performance change. Guan et al. demonstrated a photoelectric memristor with a structure of Ag/SrTiO_3_/CsPbBr_3_/Au. It was found that during the SET process, the perovskite RS characteristics demonstrate a dual‐band response to UV (365 nm) and visible light (505 nm), in which UV light exhibits a more pronounced effect to lower the set voltage, suggesting that light with higher photon energy may promote the formation of conductive filaments and reduce the energy consumption.^[^ ^78^ ^]^ In short, light coupling with higher intensity and photon energy typically reduces the required voltage to switch to LRS, enhancing the perovskite device with greater efficiency. On the other hand, reset voltage varies, where light may reduce it in some cases or remain unchanged in others, depending on the perovskite materials and device structures.

### 5.2 Improved Operation Reliability

**Figure 8:**
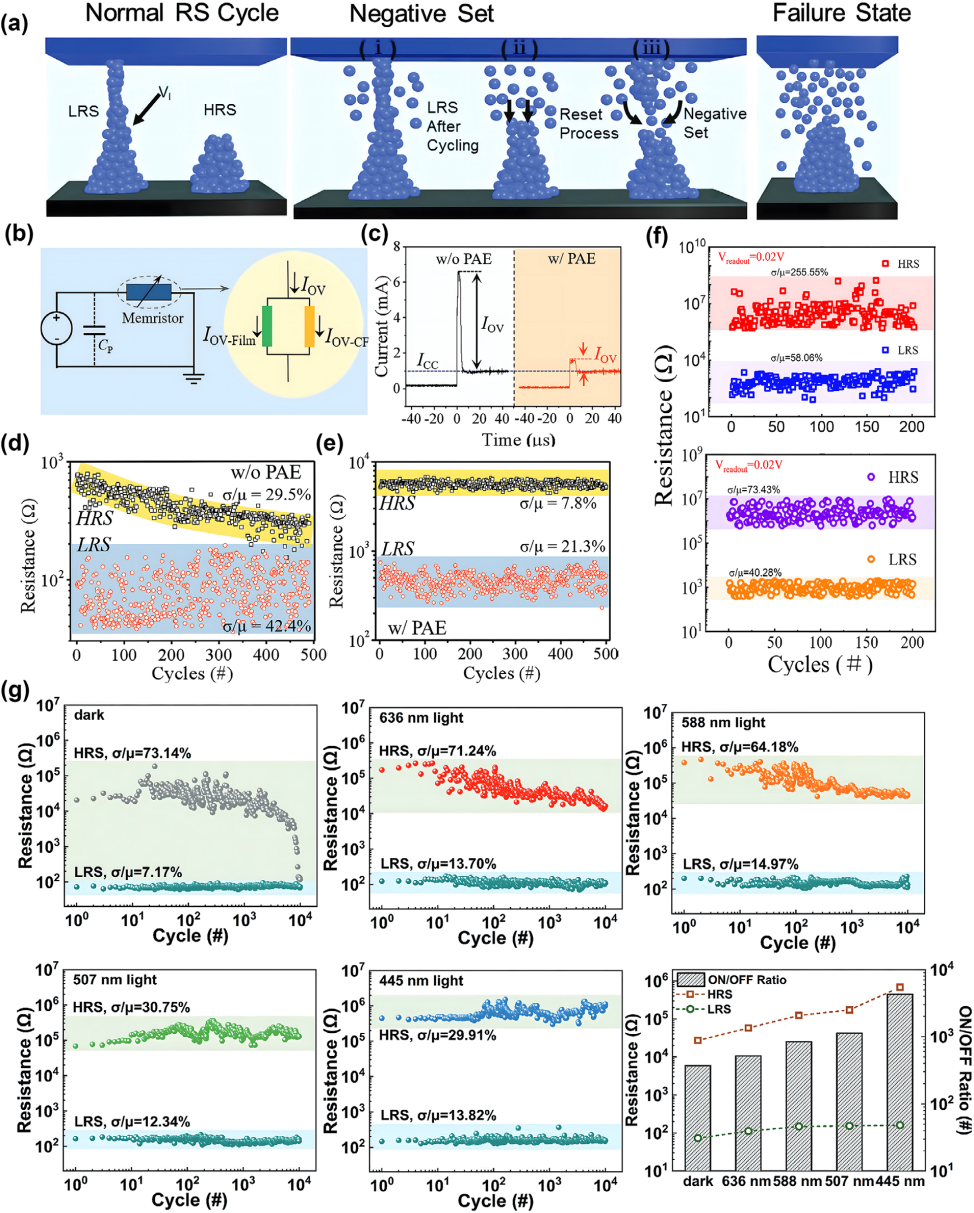
a) Schematic diagram showing the formation and rupture of conductive filament in normal set and reset cycles and failure cases, large conductive filament with complex shape. Reproduced with permission.^[^ ^80^ ^]^ Copyright 2019, Wiley‐VCH. b) Equivalent circuit diagram showing how overshoot current affects the RS device. c) Difference in overshoot current exceeding compliance current during the reset process with and without PAE method in FTO|MAPbI_3_|Al device. d,e) Improved consistency in 500 cycle endurance test for both HRS and LRS in FTO|MAPbI_3_|Al device using PAE method. (b–e) Reproduced with permission.^[^ ^76^ ^]^ Copyright 2020, Wiley‐VCH. f) Light improved consistency in FTO|Cs_2_Pb(SCN)_2_I_2_|Al device during 200‐cycle endurance tests. Reproduced with permission.^[^ ^85^ ^]^ Copyright 2023, Elsevier. g) Improved operation reliability and consistency, as well as the On/Off ratio for ITO|PEDOT:PSS|MAPbI_3_|EGaIn device under various wavelengths of light. Reproduced with permission.^[^ ^86^ ^]^ Copyright 2023, Wiley‐VCH.

In addition to reducing power consumption, light‐induced RS has been reported to improve the operation reliability of perovskite memristor devices. Typical failures in RS devices include fluctuations in the HRS and LRS current levels, as well as decreases in HRS resistance during cyclic endurance or retention tests.^[^ ^25^, ^37^ ^]^ In general, the formation of large, complex‐shaped conductive filaments during multiple RS cycles is responsible for these failures,^[^ ^80^, ^81^ ^]^ as shown in **Figure** **8**a. Additionally, large‐sized conductive filaments can result in resistance decreases in HRS, and increase power consumption during the reset process, as more energy is required to rupture these filaments.^[^ ^82^, ^83^ ^]^ Zhao et al.^[^ ^76^ ^]^ proposed that the overshoot current (*I*_OV_) during the RS cycles is the main origin of the formation of large‐sized conductive filaments, and the PAE method can suppress the negative effect of *I*_OV_ during the switching process, thereby improving device reliability. During RS device testing, the compliance current is often used to protect the device from breakdown, and ideally, the reset current should be approximately equal to the compliance current.^[^ ^84^ ^]^ The *I*_OV_ is the current that temporarily exceeds the compliance current during the switching process, as shown in Figure 8b, which should have a linear relationship with the compliance current ideally. The *I*_OV_ can flow in parallel through the device via the conductive filament and the film. Under light stimulation, the conductivity of the film increases due to photogenerated electron–hole pairs, which enhance the current flowing through the film and hence reduce the current flowing through the conductive filament.^[^ ^76^ ^]^ Thus, the light coupling can effectively prevent the overgrowth of conductive filaments, leading to improved device reliability. Figure 8c shows direct measurement of the *I*_OV_ in FTO|MAPbI_3_|Al devices, in which the device with the PAE method has a significantly lower *I*_OV_ and thus a lower chance of conductive filament overgrowth. Zhao et al.^[^ ^76^ ^]^ conducted a 500‐cycle endurance test on the FTO|MAPbI_3_|Al device, as shown in Figure 8d,e. For samples without using PAE, the On/Off ratio decreased with increased cycle numbers, while in PAE‐assisted samples, both HRS and LRS current levels increased, but the On/Off ratio remained constant over 500 cycles. The *σ*/*µ* labels represent the coefficient of variation (CV) for HRS and LRS currents, indicating the fluctuations of current levels during the cyclic test. The CV for HRS current reduced from 29.5% to 7.8% and for LRS current from 42.4% to 21.3% after introducing the light assistive forming, demonstrating the significant effect of light on improved operation reliability.

A similar result has also been observed in other light‐assistive RS devices. Li et al.^[^ ^85^ ^]^ studied the RS device based on Cs_2_Pb(SCN)_2_I_2_ perovskite, combined with FTO and Al electrode, as shown in Figure 8f. Without light irradiation, the device shows a bipolar switching with an On/Off ratio of 10^3^, and the CV for the HRS and LRS were 255.55% and 58.06%, respectively. After light irradiation, the fluctuations in HRS and LRS reduced to 73.43% and 40.28%, respectively, while the On/Off ratio remained approximately constant at 10^3^. This demonstrates a significant improvement in operation reliability induced by light. Lv et al.^[^ ^37^ ^]^ studied double perovskite Cs_2_AgBiBr_6_ (CABB) with a Pt TE and an ITO BE, using a 445 nm, 4.67 mW cm^−2^ laser to tune the RS properties. During the retention test without light, the LRS current reverted to the HRS level after 450 s under dark conditions. In contrast, under light irradiation, the HRS and LRS current levels remained stable for a 2400 s retention test, with an On/Off ratio significantly larger than that of the device in the dark. This test demonstrates that light coupling can enhance the retention property of RS devices. Liu et al.^[^ ^86^ ^]^ further studied the effect of light wavelength on the reliability of ITO|PEDOT:PSS|MAPbI_3_|EGaIn device (Figure 8g). In a dark environment, the device failed after 8000 to 10 000 cycles due to the collapse of the HRS state. Under light illumination, no failures were observed for any of the tested wavelengths. However, deterioration in the HRS state was still noted for samples exposed to 636 and 588 nm light sources. When using 507 and 445 nm light sources, no significant failure was observed in the HRS state, and fluctuations in the HRS were limited to ≈30%, demonstrating the best reliability performance.

### 5.3 Multilevel Data Storage

**Figure 9:**
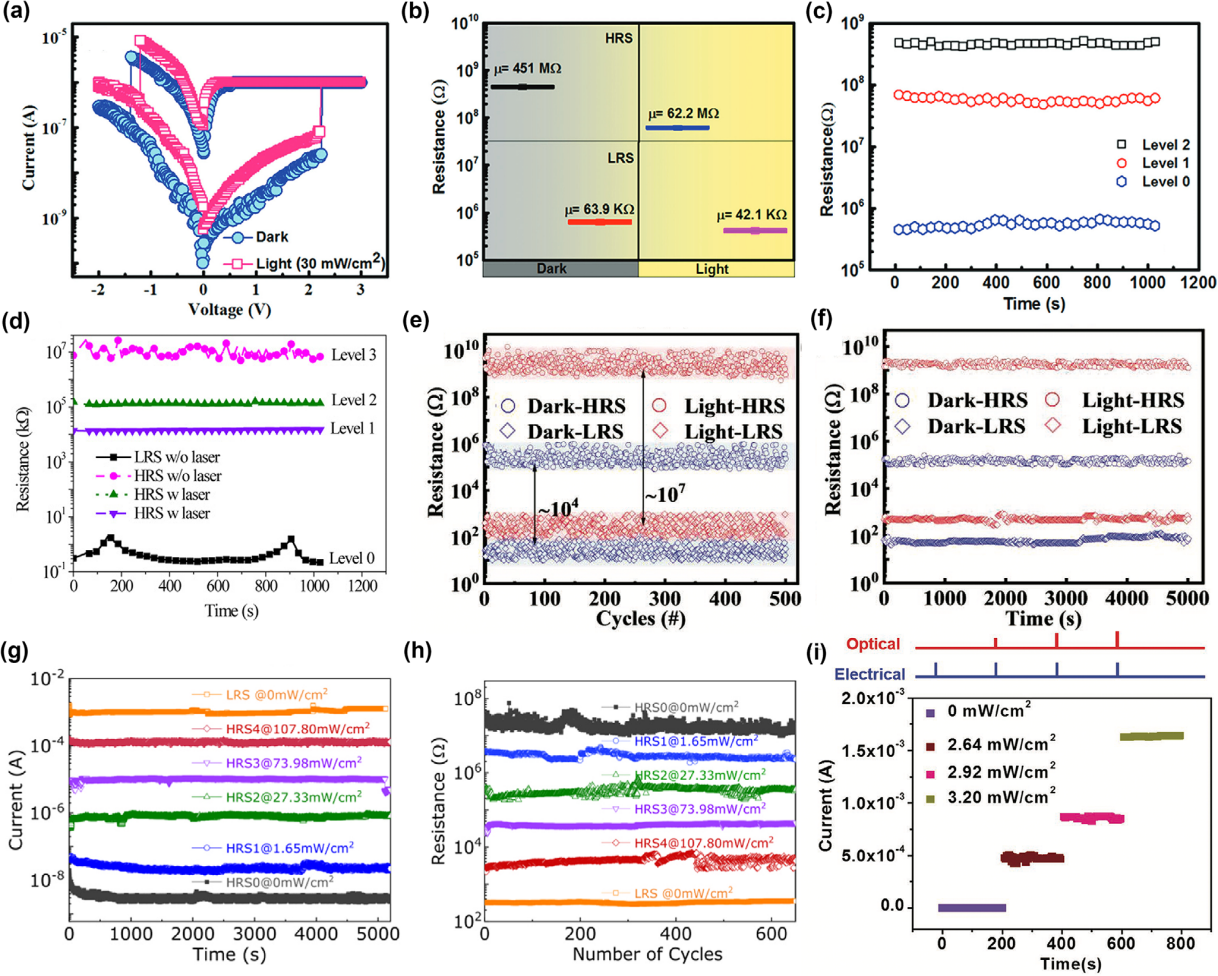
a) Different hysteresis loops of ITO–PET|CsPb_2_Br_5_|Al device under dark and light conditions, respectively. b,c) Different resistance levels at the HRS state of ITO–PET|CsPb_2_Br_5_|Al device, forming level 1 and level 2 resistances. (a–c) Reproduced with permission.^[^ ^88^ ^]^ Copyright 2022, Royal Society of Chemistry. d) Multilevel data storage ability on ITO|CsPbBr_3_|Au device achieved with 405 nm laser irradiation at 80 mW cm^−2^ (level 1), 30 mW cm^−2^ (level 2), and dark (level 3) conditions. Reproduced with permission.^[^ ^36^ ^]^ Copyright 2019, AIP Publishing. e,f) ITO|CsPbBr_3_:GO|Ag device behaving multilevel resistance in cyclic and retention test. Reproduced with permission.^[^ ^75^ ^]^ Copyright 2022, Wiley‐VCH. g,h) Multilevel resistance state achieved in ITO|MA_2−_ *_x_* Cs*_x_* ABB|Ag device, by controlling the power output of the light source, from 1.65 to 107.8 mW cm^−2^. Reproduced with permission.^[^ ^91^ ^]^ Copyright 2024, Elsevier. i) FTO|MAPbI_3−_ *_x_* Cl*_x_* |Au device behaving multilevel resistance under voltage pulse and light pulse. The voltage pulse used is 0.13 V with 100 µs duration. The duration of the light pulse is 1 s with different power intensities. The read voltage is 0.05 V. Reproduced with permission.^[^ ^15^ ^]^ Copyright 2018, Wiley‐VCH.

As mentioned in the previous section, many RS perovskite devices demonstrate different HRS and LRS current values under various conditions. In typical RS devices, this phenomenon can be observed when setting different compliance currents during the SET process,^[^ ^87^, ^88^ ^]^ which has also been used to control the retention and durability of RS devices. Similarly, in light‐induced RS devices, different current levels can be achieved by using light sources of varied wavelengths or power intensities to control the RS behavior, hence enabling the storage of multiple information states within a single memory bit. Paul et al.^[^ ^88^ ^]^ studied an ITO–PET|CsPb_2_Br_5_|Al device, which shows a typical bipolar switching under both dark and 405 nm light illumination (30 mW cm^−2^), as shown in **Figure** **9**a. The resistance of the device in the LRS state is similar between dark and light conditions, but in the HRS state, the device's resistance under dark conditions is 451 MΩ, while the resistance under light is 62.2 MΩ, which is one order of magnitude lower than dark conditions. This distinct difference in HRS resistance, combined with the LRS state, enables a three‐level switching device, as shown in Figure 9b,c. Furthermore, the resistance levels can also be controlled by using light sources with different power intensities. In 2019, Chen et al.^[^ ^36^ ^]^ proposed a CsPbBr_3_ QD RS device with Au and ITO electrodes. This device exhibits bipolar switching, and its multilevel switching behavior was investigated by testing the resistance in the HRS under varied illumination intensities. As shown in Figure 9d, the result shows three distinct HRS levels corresponding to 405 nm laser irradiation at 80 mW cm^−2^, 30 mW cm^−2^, and dark conditions, with resistance values of 10^4^, 10^5^, and 10^7^ kΩ, respectively. These resistance levels are clearly distinguishable, and all three HRS show large On/Off ratios compared to the LRS On state. These examples achieved their multilevel switching by decreasing the resistance in the HRS state due to the effect of photoinduced current. However, it comes with a drawback of a decrease in the On/Off ratio and memory window. To address this, further research has been conducted to enhance the On/Off ratio of light‐coupled RS devices for multilevel switching. In 2022, Liu et al.^[^ ^75^ ^]^ reported that a CsPbBr_3_ QD device with Ag and ITO electrodes can vary the HRS and LRS current levels under light illumination. As shown in Figure 9e,f, in both cyclic endurance testing and retention testing, the device presents different resistance states when switching the light on and off, in both HRS and LRS, which is a typical multilevel resistance behavior, and the device demonstrates a significantly larger On/Off ratio under light illumination due to the increased LRS current upon light coupling. It is noticeable that in this work, the resistance changes in LRS after light coupling are not as large as HRS, which shows significant differences. The possible reason is that in the LRS, the metallic filament dominates the conductivity, which is less responsive to photogenerated carriers. In the HRS, without the conductive filament, the semiconducting functional layer dominates the current flow, where the light absorption excites electrons into the conduction band thus greatly changing the conductivity.^[^ ^89^ ^]^ A similar multilevel switching is also achieved with CsPbBr_3_ combining with Au TE. Ray and Pal^[^ ^90^ ^]^ tested the cyclic and endurance test of this device for 400 cycles and 1000 s, respectively, and demonstrated the shift in both HRS and LRS state under a stimulated solar light source with 0.1 W cm^−2^ power density. In another work, Huang et al.^[^ ^91^ ^]^ tested ITO|MA_2−_ *_x_* Cs*_x_* ABB|Ag device under five different illumination intensities, from 0 mW cm^−^^2^ (dark condition) to 107.80 mW cm^−^^2^, as shown in Figure 9g,h. With the increase of power intensity, five different current levels in the HRS state can be clearly observed in the retention test of more than 5000 s and cyclic endurance test over 600 cycles, demonstrating a stable multilevel switching characteristic.

Besides these examples using continuous light illumination, multilevel resistance can also be achieved by appropriately coupling electrical and optical pulse signals. Zhou et al. proposed an FTO|MAPbI_3−_ *_x_* Cl*_x_* |Au device in 2018, which demonstrates bipolar switching combined with a significantly reduced SET voltage from 1.47 to 0.1 V after integrating light signals. The author tuned the resistance level by applying 0.13 V with 100 µs duration voltage pulse combined with white light optical pulses (duration 1 s, power density from 0 to 3.20 mW cm^−2^). The different resistance levels are measured by a constant reading voltage of 0.05 V and maintained 200 s for each level, as shown in Figure 9i. With the increase in power density of light, the read current gradually decreases and forms four levels of resistance at dark, 2.64, 2.92, and 3.20 mW cm^−2^, which demonstrates the ability to form multilevel storage with integrated electrical–optical pulses.

### 5.4 Nonvolatile Light Coupling in RS Devices

While coupling light into perovskite memristors offers numerous advantages, including enhanced energy efficiency, the fundamental electrical properties whether volatile or nonvolatile remain largely determined by the intrinsic characteristics of the original device. In other words, upon removal of the light stimulus, the memristor reverts to its inherent resistive RS behavior, failing to preserve the information carried by the light even in nonvolatile devices capable of storing electrical signals. Ideally, if the RS devices can retain and process light signals, it would greatly expand the range of potential applications, especially in optoelectronic neuromorphic computing and sensory integration systems. In this regard, recent studies have focused on the nonvolatile properties of perovskite RS devices when coupled with light, so‐called photomemory. Guan et al.^[^ ^92^ ^]^ employed a multilayer architecture comprising two MAPbBr_3_ perovskite layers sandwiching an Au middle layer, with an Ag TE and an ITO BE, demonstrating bipolar switching behavior and achieving stable nonvolatile RS memory under optical illumination (470 nm, 10 mW cm^−2^). The underlying concept is that the top MAPbBr_3_ layer functions as a photodetector, absorbing incident light and modulating the voltage distribution across the vertically stacked device, in turn, affecting the bottom MAPbBr_3_ layer, which serves as the RS memory. Upon illumination, the resistance of the top MAPbBr_3_ layer decreases, leading to a larger voltage drop across the bottom MAPbBr_3_ memory due to the series connection, thus triggering the Set process. Consequently, the device switched to LRS under illumination and remained in the LRS after removing the light source, clearly demonstrating nonvolatile behavior. A similar nonvolatile photomemory was observed in another study using devices with CsPbBr_3_/SrTiO_3_ heterojunction layers combined with Ag and Au electrodes.^[^ ^78^ ^]^ These devices exhibited bipolar switching with a set voltage of ≈3 V under dark conditions, reduced to ≈0.5 V under illumination with varying wavelengths (340–620 nm) and different power intensities (0.5–2 mW cm^−2^). The light illumination can lower the energy barrier of heterojunction interface for Ag ion migration, enabling partial filament formation under intermediate bias and modulating the device resistance. Upon removal of light, due to the large heterojunction barrier, the filament cluster gradually dissolves and retains within the perovskite layer contributing to their nonvolatility of photomemory. The capability of perovskite photomemory offers the unique advantage of integrating optical sensing and nonvolatile data storage within a single architecture, enabling direct light‐to‐memory signal conversion without intermediate circuitry. Additionally, their tunable bandgaps,^[^ ^93^ ^]^ high absorption coefficients,^[^ ^94^ ^]^ and solution‐processability make halide perovskite ideal for low‐cost, high‐density, and multifunctional optoelectronic memory applications.

### 5.5 Light‐Integrated Artificial Synapse

In recent years, bio‐inspired neuromorphic computing systems have been proposed aiming to overcome the issues in conventional processing systems using von Neumann architectures. This type of system contains neurons as individual computing and storage units, inspired by the nervous system in the human body. In a biological neuron, longer fibers are usually referred to as axons and shorter fibers are called dendrites. Two neurons are connected to each other with their axons and dendrites paired to each other, with a cleft found between the axon and dendrite. This structure is responsible for neuromorphic signal transmission and is usually called “Synapse,”^[^ ^7^ ^]^ and neurons can be connected in this way and forming a neuron network.^[^ ^95^ ^]^ The basic synaptic functions include potentiation and depression, corresponding to the learning and forgetting behavior. In the biological neurons, the potentiation and depression are defined by the change in weight of the synapses (Δ*w*). Potentiation includes short‐term potentiation (STP) and long‐term potentiation (LTP), which correspond to the short‐term and long‐term memory in the human brain, while in the artificial synapse, postsynaptic current (PSC), and excitatory postsynaptic current (EPSC) are widely used in to represent the synapse weight. For perovskite‐based RS devices functioning as artificial synapses, the synaptic behavior can be modulated by applying different voltage pulse parameters. The underlying principle is that when the duration of the voltage pulse train is sufficiently short, the RS device does not directly switch between the HRS and LRS, as ion migration and accumulation require time. Instead, the resistance changes gradually, mimicking the regulation of synaptic weight in biological neurons. This gradual modulation allows the synaptic behavior of perovskite RS memristors to be finely tuned by adjusting the input pulse train, including changing the number of pulses (spike number‐dependent plasticity, SNDP), the gap between pulses (spike time‐dependent plasticity, STDP), the pulse duration (spike duration‐dependent plasticity, SDDP), and the amplitude (spike intensity‐dependent plasticity, SIDP) of the voltage pulse.^[^ ^96^ ^]^

**Figure 10:**
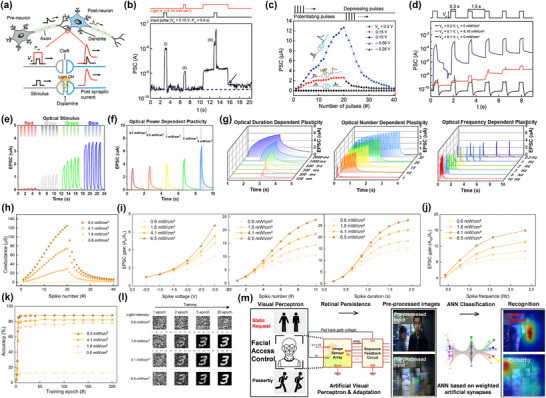
a) Schematic diagram showing the synapse diagram under light stimulation. b) PSC response in ITO|MAPbI_3_|Ag device under (i) voltage pulse, (ii) light pulse, and (iii) combined light and voltage pulse. c) Potentiation and depression behavior under different voltage pulses and light pulses. d) STP and LTP behavior. (a–d) Reproduced with permission.^[^ ^26^ ^]^ Copyright 2019, Wiley‐VCH. Li–AlO*_x_* |InO|FAPbI_3_|ZnO device's EPSC response to the optical pulses with e) different wavelengths, f) different intensities, and g) optical pulse duration, pulse number, and frequency. (e–g) Reproduced with permission.^[^ ^101^ ^]^ Copyright 2022, Elsevier. FAPbI_3_ perovskite hybrid with MA groups showing h) potentiation and depression under different light intensities. i) EPSC gain dependence on voltage pulse amplitude, pulse number, and pulse duration, under different light intensities. j) EPSC gain dependent on pulse frequency. k) Visual recognition accuracy under different light intensities for 200 stimulated epochs. l) Schematic diagram showing visual recognition stimulation. (h–l) Reproduced with permission.^[^ ^102^ ^]^ Copyright 2021, Elsevier. m) Schematic diagram showing ANN formed by light‐integrated synapse used in visual perception. Reproduced with permission.^[^ ^101^ ^]^ Copyright 2022, Elsevier.

For light‐coupled artificial synapse devices, synaptic functions and weight regulation can be achieved by applying optical pulse input or integrating voltage pulses with optical signals, as shown in **Figure** **10**a. This not only enhances the functionality and energy efficiency of synapse devices due to the lowering of operation voltage but also enables the opportunity for processing the light signals directly. The light pulse can act as an external stimulus apart from voltage pulses, which makes optoelectronic logic operations and artificial vision systems possible.^[^ ^97^ ^]^ For example, Liu et al. demonstrated that Cs_2_AgBiBr_6_‐based synapse devices can be programmed both electronically and optically, achieving synaptic plasticities such as LTP and STP. It also realized paired‐pulse facilitation (PPF) under both electric and light stimuli. The PPF index is calculated with the following equation, where *A*_1_ and *A*_2_ refer to the EPSC peak, respectively, *C*_1_ and *C*_2_ refer to the initial facilitation amplitudes, and *τ*_1_ and *τ*_2_ refer to relaxation time constants^[^ ^98^ ^]^

(1) $$\mathrm{PPF}\;\mathrm{index}\;=\frac{A_{2}}{A_{1}}=1+C_{1}e^{-\frac{\Delta t}{\tau_{1}}}+C_{2}e^{-\frac{\Delta t}{\tau_{2}}}$$

In 2019, Ham et al.^[^ ^26^ ^]^ fabricated an ITO|MAPbI_3_|Ag device that exhibits bipolar switching and investigated the PSC of the device, corresponding to the synaptic weight, as the output signal. The author investigated the modulation of PSC using electric signals, light signals, and a combination of both, as shown in Figure 10b. A PSC current at the 10^‐−8^ A level (peak (i)) was recorded with an input pulse of 0.15 V amplitude and 0.4 s pulse width, and a PSC current at the 10^−9^ A level (peak (ii)) was observed when introducing 8.16 mW cm^−2^ light. When integrating the electric pulse with the light source, a significantly larger PSC output was observed, surpassing the peaks of individual electric (i) and light (ii) signals. This study indicates that light can effectively modulate synaptic plasticity, either independently or in combination with an electrical signal. The potentiation and depression synaptic functions were also achieved using the device, as shown in Figure 10c, in which under the same voltage of 0.15 V, the device behaves no synaptic behavior under dark conditions, but can potentiate at 0.15 V with 8.16 mW cm^−2^ light pulse.

Ham et al.^[^ ^26^ ^]^ further investigated the conditions for STP and LTP using the perovskite device, as shown in Figure 10d. When using light pulses alone, only STP behavior was observed, regardless of the light pulse frequency, duration, and intensities tested. For electrical input, when using electrical signals alone, LTP behavior was observed with a large amplitude of 0.3 V, as indicated by the blue line in the figure. When using a smaller electrical input with 0.1 V, the device only exhibits STP, however, the device transitioned to LTP when the 0.1 V voltage pulse was integrated with an 8.16 mW cm^−^^2^ light pulse. This suggests that while the photogenerated current alone may be insufficient to sustain a robust conductive filament, light modulation can still reduce the barrier for ion migration and lower the threshold for LTP.^[^ ^62^, ^99^ ^]^ This reduction in threshold shows the potential for energy‐efficient neuromorphic computing systems and also simulates the dopamine‐facilitated synapse activity.^[^ ^100^ ^]^

In 2022, Liu et al.^[^ ^101^ ^]^ studied the EPSC response of Li–AlO*_x_* |InO|FAPbI_3_|ZnO device. The device showed different EPSC currents when applying light pulses with different wavelengths and intensities, as shown in Figure 10e,f. Similar to the human eye that generates varied synaptic weights in response to different light stimuli, the EPSC weight of the device changes when exposed to red, white, green and blue light, and the weight increases as the light power density rises from 0.2 to 3 mW cm^−2^, demonstrating optical power dependent plasticity. 3D charts in Figure 10g demonstrate the plasticity dependent on the duration, number, and frequency of light pulses, proving this device features excellent optical synaptic behavior. Similar studies were conducted by Gong et al.^[^ ^102^ ^]^ on a device based on FAPbI_3_ perovskite hybrid with MACl and MAI groups, and combining with Au and ITO electrodes. As shown in Figure 10h, the device behaves potentiation and depression more significantly when increasing the intensity of light, revealing a superior light coupling effect. Gong et al.^[^ ^102^ ^]^ further studied the SIDP, SNDP, SDDP, and STDP behaviors of the device under different intensities of light. Figure 10i plots the gain of EPSC, corresponding to the change of synapse weight, versus voltage pulse intensity, number, and duration, while Figure 10j displays the EPSC gain versus different pulse frequencies. The device behaves all of these synapse functions more intensely with the increase of light source power intensity. Based on the potentiation and depression result of the perovskite synaptic device, Gong et al.^[^ ^102^ ^]^ examined its usage in handwritten pattern recognition using the artificial neuron network (ANN). As shown in Figure 10k, after 200‐epoch training, the pattern recognition accuracy reached the highest of 88.2% when the device was subjected to 6.5 mW cm^−2^ illumination. The light intensity‐dependent training, shown in (Figure 10l), reveals that recognition accuracy declines with reduced light intensity, in which the device under 1.8 mW cm^−2^ of light can only reach ≈70% accuracy, and the device under 0.6 mW cm^−2^ illumination failed to provide accurate recognition. Liu et al.^[^ ^101^ ^]^ also applied their FAPbI_3_ RS devices for facial recognition, as shown in Figure 10m. The optical duration‐dependent plasticity of this device allows it to distinguish a static target from moving passersby in the background, demonstrating excellent performance in visual recognition and perceptron functionality.

## 6 Summary and Outlooks

This review has comprehensively discussed the mechanisms and advantages associated with HPs‐based light‐assisted RS devices. The distinctive optoelectronic properties of HPs, including their tunable bandgaps and superior charge transport capabilities, have positioned these materials as promising candidates for next‐generation memory and neuromorphic computing technologies. The capability to integrate light signals has been demonstrated to play a pivotal role in enhancing the performance of RS devices, primarily by inducing the generation of electron–hole pairs and facilitating ionic movement/accumulation. Moreover, light‐assisted RS devices offer several notable advantages, including reduced power consumption, improved device reliability, capability in multilevel data storage, and the potential for artificial synapse application. The reduction in switching voltage, enabled by photogenerated carriers and ionic transport adjustments, provides a viable pathway for developing low‐energy memory devices, while the enhanced operation reliability ensures better endurance and retention during cyclic operations. Additionally, the capacity for achieving multilevel resistance states, driven by varying light intensities and wavelengths, presents a promising opportunity for high‐density data storage applications. The light‐responsiveness of these devices opens new frontiers in artificial synapse development, particularly for neuromorphic computing systems that aim to mimic biological synaptic functions. By adopting the electrical–optical integration strategy, the HP‐based RS synapse devices can not only be trained and learned more efficiently with reduced power consumption but also enable the processing of light signals directly, showing great promise for the development of advanced neuromorphic computing systems.

Despite the achievements accomplished in recent decades, the advancement of light‐induced RS devices hinges on overcoming critical challenges in material selection, durability, stability, and integration. Future research should focus on but not limited to the following key points.

1. Despite we mentioned some lead‐free HP examples in this review, the most high‐performance HP materials are still lead‐based. The dominant use of lead‐based HPs in light‐induced RS devices raises significant environmental and health concerns due to lead toxicity. Future research should prioritize the development of lead‐free alternatives with comparable or superior optoelectronic properties.
2. Metal electrode corrosion in halide perovskite devices poses a significant challenge due to halide ion migration, redox reactions with metals like Ag or Al, and environmental factors like moisture and light, which degrade both the electrode and perovskite layer.^[^ ^103^, ^104^, ^105^, ^106^, ^107^ ^]^ Research efforts have focused on interface engineering with buffer layers (e.g., BCP, MoO_3_, SnO_2_) to block ion diffusion,^[^ ^108^, ^109^ ^]^ adopting corrosion‐resistant carbon electrodes,^[^ ^110^, ^111^ ^]^ and using stable metals or alloys like AgAl with protective oxides.^[^ ^112^ ^]^ Additional strategies include stabilizing the perovskite through compositional tuning or additives to limit halide release,^[^ ^113^ ^]^ developing metal‐free designs with conductive polymers like PEDOT:PSS,^[^ ^106^ ^]^ and enhancing encapsulation to mitigate environmental effects. Stability studies further reveal corrosion dynamics under operational conditions, emphasizing charge and defect roles.^[^ ^114^, ^115^ ^]^ While progress is notable, balancing stability, efficiency, and scalability remains a key focus for future commercialization.
3. In this review, we discussed improved operation reliability by introducing light illumination during the switching process, which facilitates stable operation by controlling the overshoot current. However, the fragility of perovskite materials, particularly their vulnerability to moisture, oxygen, and thermal degradation, still represents a substantial barrier to long‐term device stability. Studies indicated that perovskite‐based device has lower On/Off ratio (some may have higher On/Off ratio at beginning) and unstable RS behavior under humid condition.^[^ ^116^, ^117^ ^]^ Efforts must focus on improving the inherent stability of perovskites through chemical and structural modifications. As the long‐term stability of perovskite solar cells has continued to improve significantly in recent years, similar strategies can be adopted for perovskite RS memristors to enhance their long‐term usage.
4. The migration of ions is influenced by the perovskite's crystal structure, defects, and applied electric fields. The drift model describes ion movement under an electric field, since under an applied electric field, ions move directionally, creating or disrupting conductive paths.^[^ ^118^ ^]^ On the other hand, the diffusion model accounts for random thermal motion driven by concentration gradients. It contributes to the spreading of ions, affecting the stability and uniformity of switching. This is critical in volatile dynamics, where diffusion‐based behavior ensures short‐term memory functions.^[^ ^119^ ^]^ Together, these mechanisms govern the formation and rupture of conducting filament. The integration of the drift and diffusion model aids in designing perovskite memristors with specific characteristics, such as fast switching and high robustness, as noted in image processing applications, while also addressing challenges like hysteresis and environmental stability.^[^ ^120^, ^121^ ^]^ However, challenges persist, including quantifying the exact contributions of drift versus diffusion under varying conditions and ensuring long‐term stability, necessitating validation against experimental data, as seen in studies comparing simulated *I*–*V* curves with measured responses.^[^ ^122^ ^]^ Future research could refine the model to include temperature effects, as suggested in compact models,^[^ ^123^ ^]^ potentially leading to perovskite memristors with enhanced performance for artificial synapses and neural networks, aligning with the growing demand for brain‐inspired computing.
5. RRAM devices can achieve high‐density packing with crossbar matrix structures. However, integrating precisely controlled continuous or pulsed light illumination mechanisms within such structures remains a significant challenge that requires further exploration. While the promising future of light‐integrated RS devices for ANNs and neuromorphic computing, most published studies still rely on simulations rather than physical devices. To bridge this gap, future research should prioritize the fabrication of physical devices that emulate artificial synaptic behavior. Efforts should focus on integrating RS devices into functional ANN architectures, addressing challenges such as precise control of synaptic weight modulation and device uniformity.
6. The unique properties and soft lattice of perovskites make them ideal candidates for transparent, flexible, and wearable electronics. Current research has already made significant progress in integrating flexible and transparent electrodes into RS devices, demonstrating their potential for multifunctional applications. These advancements pave the way for RS devices to operate as integrated sensors, displays, and memory components within wearable systems. To further expand the utility of light‐integrated perovskite RS devices, future research should focus on exploring their applications in flexible electronics and smart textiles. Such innovations could redefine the capabilities of memory devices, enhancing their form factor and functionality to meet the demands of emerging technologies.

By advancing research in these areas, we can further unlock opportunities for light‐coupled HP RS devices for low‐power, high‐performance, and versatile optoelectronic data storing and neuromorphic computing applications in future technologies.

## Conflict of Interest

The authors declare no conflict of interest.
